# VEGF-A/NRP1 stimulates GIPC1 and Syx complex formation to promote RhoA activation and proliferation in skin cancer cells

**DOI:** 10.1242/bio.010918

**Published:** 2015-07-24

**Authors:** Ayumi Yoshida, Akio Shimizu, Hirotsugu Asano, Tetsuya Kadonosono, Shinae Kizaka Kondoh, Elena Geretti, Akiko Mammoto, Michael Klagsbrun, Misuzu Kurokawa Seo

**Affiliations:** 1 Division of Engineering (Biotechnology), Graduate School of Engineering, Kyoto Sangyo University, Kyoto 603-8555, Japan; 2 Department of Molecular Biosciences, Faculty of Life Sciences, Kyoto Sangyo University, Kyoto 603-8047, Japan; 3 Biofunctional Engineering, Graduate School of Bioscience & Biotechnology, Tokyo Institute of Technology, Tokyo 226-8503, Japan; 4 Vascular Biology Program, Boston Children's Hospital, Departments of Surgery andPathology and Harvard Medical School, Boston, MA 02115, USA

**Keywords:** Cancer, VEGF-A, Neuropilin, Syx, RhoA

## Abstract

Neuropilin-1 (NRP1) has been identified as a VEGF-A receptor. DJM-1, a human skin cancer cell line, expresses endogenous VEGF-A and NRP1. In the present study, the RNA interference of VEGF-A or NRP1 suppressed DJM-1 cell proliferation. Furthermore, the overexpression of the NRP1 wild type restored shNRP1-treated DJM-1 cell proliferation, whereas NRP1 cytoplasmic deletion mutants did not. A co-immunoprecipitation analysis revealed that VEGF-A induced interactions between NRP1 and GIPC1, a scaffold protein, and complex formation between GIPC1 and Syx, a RhoGEF. The knockdown of GIPC1 or Syx reduced active RhoA and DJM-1 cell proliferation without affecting the MAPK or Akt pathway. C3 exoenzyme or Y27632 inhibited the VEGF-A-induced proliferation of DJM-1 cells. Conversely, the overexpression of the constitutively active form of RhoA restored the proliferation of siVEGF-A-treated DJM-1 cells. Furthermore, the inhibition of VEGF-A/NRP1 signaling upregulated p27, a CDK inhibitor. A cell-penetrating oligopeptide that targeted GIPC1/Syx complex formation inhibited the VEGF-A-induced activation of RhoA and suppressed DJM-1 cell proliferation. In conclusion, this new signaling pathway of VEGF-A/NRP1 induced cancer cell proliferation by forming a GIPC1/Syx complex that activated RhoA to degrade the p27 protein.

## INTRODUCTION

Malignant tumors express vascular endothelial growth factor A (VEGF-A), a glycoprotein that recruits blood vessels, thereby supplying tumors with the oxygen and nutrients that promote tumor cell migration, proliferation, survival, permeability, and metastasis (Potente et al., 2011). VEGF-A signaling involves via two tyrosine kinase receptors, VEGFR1 and VEGFR2. A previous study demonstrated that the blockade of VEGF-A by Avastin, an antibody, or blockade of VEGFR2 with a specific kinase inhibitor, such as Sutent, suppressed tumor angiogenesis (Potente et al., 2011).

Avastin, in combination with chemotherapy, has exhibited some efficacy in clinical trials for metastatic colorectal cancer, non-small cell lung cancer, renal cell carcinoma, and metastatic breast cancer (Herbst and Sandler, 2008; Mountzios et al., 2014). However, its impact on overall survival is not well documented.

Neuropilin-1 (NRP1) is a 130 kDa transmembrane protein that has been identified as a novel VEGF-A receptor (Soker et al., 1998). NRP1 is expressed by endothelial cells and functions as a co-receptor of VEGFR2, enhancing VEGF-A binding to its receptor and promoting downstream signaling, e.g. the MAPK pathway (Kawamura et al., 2008). NRP1 is associated with tumor progression; it is strongly expressed in lung, brain, colon, ovarian, and prostate cancers with poor patient prognoses (Geretti et al., 2008). A Phase III study to evaluate the combined effects of Avastin and chemotherapy in patients with advanced gastric cancer reported that overall survival was worse in patient groups that strongly expressed tumor NRP1 than in patients with low baseline expression levels (Van Cutsem et al., 2012), suggesting that NRP1 is tumorigenic.

Structurally, NRP1 has two extracellular domains, a1a2 and b1b2, that bind SEMA3s and VEGF, respectively, in addition to a dimerization domain, transmembrane domain, and short cytoplasmic region (Geretti et al., 2008). Since NRP1 lacks kinase activity, there has been a concerted effort to elucidate the mechanisms underlying NRP1 signaling. NRP1 possesses a short cytoplasmic region of 44 amino acids that is involved in signaling. To date, the expression of NRP1 by tumor cells has been shown to contribute to proliferative signal transduction from VEGF-A. In renal cell carcinoma, the VEGF-A/NRP1 signal was found to activate Ras and promote tumor growth *in vivo* (Cao et al., 2012), while VEGF-A/NRP1 signals induced the phosphorylation of Akt leading to breast cancer cell survival (Bachelder et al., 2001). However, the precise mechanisms responsible for molecular interactions with the NRP1 cytoplasmic region remain unknown.

NRP1 lacking the C-terminus three amino acids [Ser-Gln-Ala (ΔSEA)] led to impaired vasculogenesis in zebrafish (Wang et al., 2006) and abnormal vascular remodeling during retinal development in mice (Fantin et al., 2011). A previous study showed that NRP1ΔSEA did not induce medulloblastoma tumorigenesis (Snuderl et al., 2013). NRP1 appears to signal via the SEA region.

GIPC1 (GAIP interacting protein C terminus), a scaffold protein, is the first molecule that was shown to interact with the NRP1 cytoplasmic region (Cai and Reed, 1999; Wang et al., 2010). It has a PDZ domain that binds to the SEA of NRP1 (Ballmer-Hofer et al., 2011; De Vries et al., 1998). GIPC1 is overexpressed in breast and pancreatic tumors and promotes tumor proliferation, survival, and metastasis (Chittenden et al., 2010; Muders et al., 2009; Wu et al., 2010); however, its functions have yet to be determined in detail (Muders, 2011). Syx was identified as a GIPC1 binding protein by a yeast two-hybrid system (Gao et al., 2000; Garnaas et al., 2008). Syx was found to bind to the GIPC1 PDZ domain via its C-terminus amino acids (Liu and Horowitz, 2006). It has a RhoGEF domain and activates a Rho family GTPase, specifically, RhoA. Previous studies demonstrated that Syx was expressed in vascular endothelial cells, neuronal cells, and some tumors, such as glioma cells (De Toledo et al., 2001; Liu and Horowitz, 2006; Nessling et al., 2005). RhoA drives the cell cycle into the S-phase (Croucher et al., 2010). RhoA has been implicated in virtually all stages of cancer progression. It may play a role during tumor cell proliferation and survival; for example, *in vitro*, constitutively active RhoA was shown to stimulate transformation (Vega and Ridley, 2008). The activation of RhoA is known to induce the protein degradation of p27^kip1^, a cyclin-dependent kinase inhibitor (CDI), in the G1 phase, which progresses the cell cycle, resulting in proliferation (Hu et al., 1999; Mammoto et al., 2004).

In the present study, we showed that VEGF-A promoted tumor cell proliferation via the NRP1 signaling pathway. The NRP1 cytoplasmic region was found to be essential for the transduction of VEGF-A signaling, which enhanced the interaction with GIPC1. GIPC1 subsequently formed a complex with Syx. This complex formation activated the RhoGEF activity of Syx, which led to the activation of RhoA. The downstream signaling of RhoA induced p27 protein degradation, leading to S phase entry of the cell cycle, resulting in cancer cell proliferation. A treatment with a cell-penetrating peptide designed to inhibit interactions between GIPC1 and Syx suppressed the activation of RhoA as well as cancer cell proliferation.

In summary, we proposed a novel signal transduction pathway of VEGF-A/NRP1 that induced cancer cell proliferation by forming a GIPC1/Syx complex that activated RhoA and degraded p27.

## RESULTS

### Knockdown of endogenous VEGF-A expression decreased human skin cancer cell proliferation *in vitro*

**Fig. 1.**
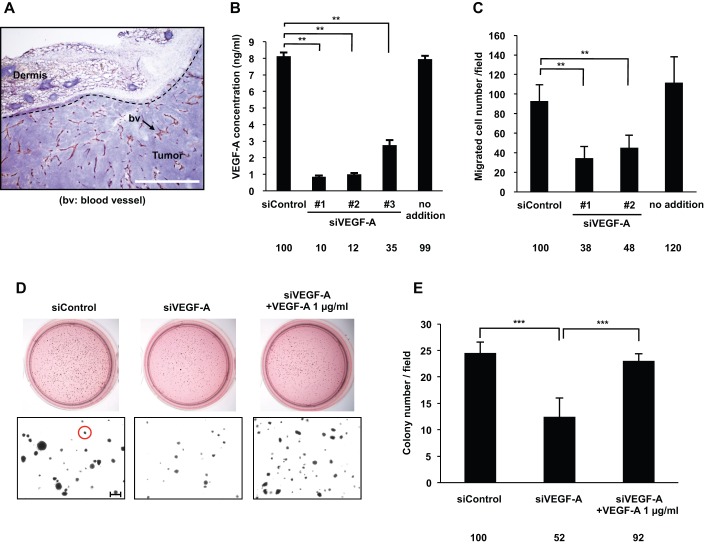
**VEGF-A secreted by DJM-1 cells induced tumor angiogenesis and cancer cell proliferation.** (A) Frozen sectioned DJM-1 tumors were stained with the endothelial marker CD-31 and hematoxylin. The arrow indicates blood vessels (Scale bar: 100 μm). (B) Quantification of VEGF-A concentrations secreted by DJM-1 cells. After a 72 h treatment with (20 nM, siControl, or siVEGF-A #1–3) or without siRNA (no addition), conditioned media were collected and analyzed by VEGF-A ELISA. (C) HUVEC migration assay. (B,C) Data represent the means±s.d. Percentages from the each mean relative to siControl are indicated below the graph. (D) Endogenous VEGF-A induced colony formation by cancer cells. DJM-1 cells were treated with siControl or siVEGF-A #1 (20 nM each) and seeded in soft agar. The upper panel shows the bright field of MTT staining colonies; the lower panel shows magnified colonies (Red circle: >80 μm diameter, Scale bar: 250 μm). (E) Quantitative analysis of D. The means of colony numbers in 6 fields for each condition are shown with ±s.d. Percentages from each mean relative to the siControl are indicated below the graph. **P<0.005; ***P<0.001.

The DJM-1 cell line was established from a human skin cancer obtained from a patient who died from metastases to the axillary lymph nodes and lungs. DJM-1 cells were orthotopically inoculated into the backs of mice. After 2 weeks, mice were sacrificed and the tumors were isolated. Tumor sections were stained with an anti-CD31 antibody (Arrow: bv) and hematoxylin. The tumors and peritumoral area were highly vascularized (Fig. 1A). The amount of VEGF-A secreted into the DJM-1 cell conditioned media (CM) was 8 ng/ml, as measured by ELISA, while that secreted into the siControl-treated DJM-1 cell CM was 7.5 ng/ml (Fig. 1B). VEGF-A was suppressed by the knockdown using 3 different siRNAs, with siVEGF-A #1 being the most effective (siVEGF-A #1: 90% inhibition, siVEGF-A #2: 88% inhibition, siVEGF-A #3: 65% inhibition, respectively) (Fig. 1B). VEGF-A secreted by DJM-1 cells stimulated the migration of HUVEC. The knockdown of VEGF-A expression suppressed the migration of HUVEC (siVEGF-A #1: 38% and siVEGF-A#2: 48% of siControl, respectively) (Fig. 1C). Colony formation in soft agar indicated cancer proliferation under anchorage-independent conditions. The knockdown of VEGF-A expression suppressed the anchorage-independent proliferation (52% of siControl) of DJM-1 cells themselves (Fig. 1D,E). The addition of exogenous VEGF-A (1 µg/ml) restored the proliferation of siVEGF-A-treated DJM-1 cells to a similar level to that of the siControl-treated cells (siVEGF-A#1+1 µg/ml VEGF-A, 92% of siControl). These results suggested that the endogenous expression of VEGF-A stimulated the proliferation of DJM-1 cells in an autocrine manner.

### VEGF-A-induced DJM-1 cell proliferation did not depend on VEGFR1 or VEGFR2

**Fig. 2.**
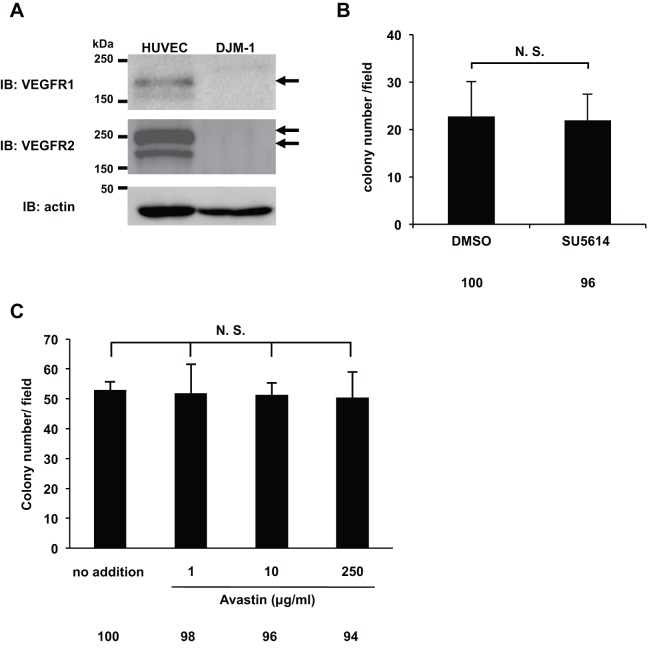
**The VEGFR kinase inhibitor SU5614 and Avastin did not inhibit DJM-1 cell proliferation.** (A) Western blot for VEGFR1 or VEGFR2 of DJM-1 cell lysates. As a positive control, the cell lysates of HUVEC were applied in the left lanes. Arrows indicate VEGFR1 or VEGFR2. (B) Colony formation assay for DJM-1 cells treated with 10 µM SU5614, the VEGFR kinase inhibitor, and with 0.2% DMSO as the control. (C) DJM-1 cell colony formation assay treated with Avastin (from 1 to 250 µg/ml). These data represent the means±s.d. N.S., not significant. Percentages from each mean relative to the DMSO (B) or no addtion (C) are shown below the graph.

VEGF-A has multiple receptors: VEGFR1, VEGFR2, and neuropilins 1 and 2 (Ferrara, 2009). The expression of VEGFR1 and VEGFR2 was detected by western blotting in HUVEC, but not in DJM-1 cells (Fig. 2A). In order to determine whether VEGFR1 or VEGFR2 signaling occurred in DJM-1 cells in response to VEGF-A, the effects of SU5614, a VEGFR tyrosine kinase inhibitor, were examined in DJM-1 cells in soft agar (Fig. 2B). However, SU5614 did not inhibit the proliferation of DJM-1 cells (DMSO: 100%, SU5614: 96%). Avastin is an antibody that neutralizes VEGF-A and targets VEGFR-binding sites. However, Avastin did not inhibit DJM-1 cell proliferation (no addition: 100%, 1 µg/ml: 98%, 10 µg/ml: 96%, 250 µg/ml: 94%, respectively) (Fig. 2C). These results suggested that autocrine VEGF-A induced cancer proliferation, but did not mediate the VEGFR1 or VEGFR2 signaling pathway.

### VEGF-A promoted cancer cell proliferation via NRP1 in an autocrine manner

**Fig. 3.**
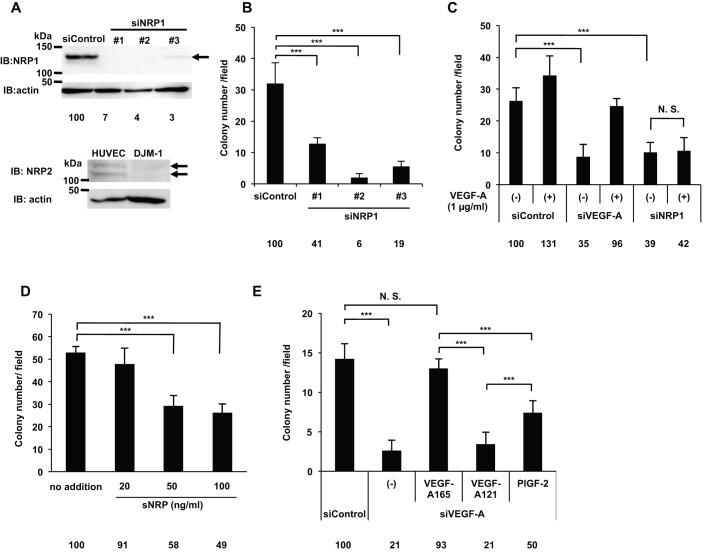
**VEGF-A promoted DJM-1 cell proliferation via NRP1 in an autocrine manner.** (A) A western blot shows that DJM-1 cells expressed NRP1, but not NRP2. DJM-1 cells were treated with siRNA (siControl, siNRP1 #1–3, 20 nM each) for immunoblotting the NRP1 protein (arrow indicates NRP1; 130 kDa). Percentages from each blotted protein amount relative to the siControl are indicated below each lane. Actin was immunoblotted to normalize the amounts of NRP1 (upper panel). HUVEC expressed NRP2, whereas DJM-1 cells did not (arrows indicate NRP2; 120–130 kDa, lower panel). (B) DJM-1 cell colony formation assay. Cells treated with 20 nM siControl and siNRP1 #1–3. (C) Colony formation by siVEGF-A- or siNRP1-treated DJM-1 cells. The presence or absence of exogenous VEGF-A (1 µg/ml) was indicated as (+) and (−) respectively. (D) DJM-1 cell colony formation assay in the presence of sNRP (from 20 to 100 ng/ml). (E) The graph shows the effects of VEGF-A family members (1 µg/ml each) in the siVEGF-A-treated DJM-1 cell colony formation assay. These data represent the means±s.d. Percentages from each mean relative to the siControl (B,C,E) or no addition (D) are shown below the graph. ***P<0.001; N.S., not significant.

DJM-1 cells express NRP1, but not NRP2. In addition, NRP1 siRNA (siNRP1) #1–3 almost completely abrogated protein expression (siNRP1 #1: 7%, #2: 4%, #3: 3% respectively), inhibiting DJM-1 cell anchorage-independent proliferation from 59 to 94% (Fig. 3A,B). Since siNRP1 #2 was the most effective inhibitor of proliferation, it was used in subsequent experiments. The siNRP1 treatment inhibited the proliferation of DJM-1 cells, similar to siVEGF-A (siControl: 100%, siNRP1: 39%, siVEGF-A: 35%, respectively) (Fig. 3C). The addition of exogenous recombinant VEGF-A did not rescue siNRP1-treated DJM-1 cell proliferation (42%), but did rescue siVEGF-A-treated DJM-1 proliferation (96%) (Fig. 3C). We also assessed the expression of the NRP1 protein by western blotting and VEGF-A by ELISA in other human cancer cell lines: PC3M, prostate cancer and U87MG, glioblastoma. The NRP1 protein (∼130 kDa) was strongly expressed in PC3M and U87MG (supplementary material Fig. S1A). All cell lines expressed NRP1, but did not express VEGFRs. U87MG cells expressed NRP1 and NRP2 (supplementary material Fig. S1A). U87MG cells secreted the highest levels of VEGF-A into conditioned medium, as shown in supplementary material Fig. S1B. The siVEGF-A or siNRP1 treatment inhibited the proliferation of PC3M (siControl: 100%, siVEGF-A: 15%, siNRP1: 23%) and U87MG cells (siControl: 100%, siVEGF-A: 33%, siNRP1: 41%) (supplementary material Fig. S1C). The addition of exogenous VEGF-A rescued the proliferation of siVEGF-A-treated cells (PC3M: 77%, U87MG: 78%). In contrast, the addition of VEGF-A did not recover the proliferation of siNRP1-treated cells (PC3M: 38%, U87MG: 46%), suggesting that NRP1 mediated VEGF-A signaling to induce PC3M and U87MG cell proliferation as in DJM-1 cells (supplementary material Fig. S1C).

Soluble-NRP (sNRP) is a VEGF-TRAP that consists of the NRP1 extracellular B domain, which is the NRP1 domain responsible for VEGF-A-binding via its exon 7- and 8-encoded regions (Parker et al., 2012). sNRP inhibited DJM-1 cell proliferation in a dose-dependent manner (20 ng/ml: 9% inhibition of no addition, 50 ng/ml: 42%, 100 ng/ml: 51%, respectively) (Fig. 3D). NRP1 bound VEGF-A_165_, but not VEGF_121_ because VEGF-A_121_ lacked the exon 7-encoded region. The addition of VEGF-A_121_ did not promote siVEGF-A-treated DJM-1 cell proliferation. PlGF-2, a member of the VEGF family that has been shown to bind NRP1 (Mamluk et al., 2002), promoted siVEGF-A-treated DJM-1 cell proliferation to 50% of siControl (siControl: 100%, siVEGF-A: 21%, siVEGF-A +VEGF-A_165_: 93%, siVEGF-A +VEGF-A_121_: 21%, siVEGF-A +PlGF-2: 50%, respectively) (Fig. 3E). These results suggested that NRP1 mediated VEGF-A signaling to promote DJM-1 cell proliferation.

### The NRP1 cytoplasmic region was responsible for VEGF-A-induced proliferation of DJM-1 cells

**Fig. 4.**
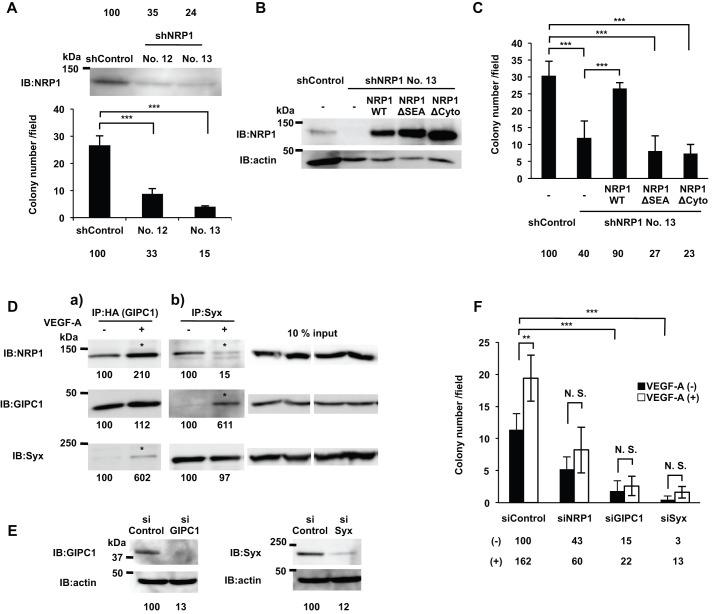
**The NRP1 cytoplasmic region was essential for VEGF-A-induced cancer cell proliferation.** (A) The western blot shows the expression of NRP1 in shControl- or NRP1 shRNA-treated DJM-1 clones (No. 12 and No. 13). (B) The comparison of NRP1 expression levels among lentivirus-overexpressed NRP1WT or cytoplasmic region deletion mutants (NRP1ΔSEA or NRP1ΔCyto) in shNRP1 DJM-1 No.13 clone (upper lanes). The same proteins were re-immunoblotted with an anti-actin antibody (lower lanes). (C) The colony formation assay of the lentivirus-overexpressed NRP1WT, NRP1ΔSEA or NRP1ΔCyto in the shNRP1 DJM-1 No.13 clone and shControl DJM-1 clone. (A,C) Percentages from each mean relative to the shControl are shown below the graph. (D) Co-immunoprecipitation assay with GIPC1 (a) or Syx (b). NRP1, GIPC1, and Syx were expressed in HEK293T cells and treated without (−) or with (+) VEGF-A (100 ng/ml) for 15 min. (a) Increased NRP1/GIPC1 and GIPC1/Syx interactions in the presence of VEGF-A are indicated by asterisks. (b) The Syx/GIPC1 interaction was increased in the presence of VEGF-A (asterisk). On the other hand, the NRP1/Syx interaction was decreased (asterisk). A 10% input as the loading control of NRP1, GIPC1, and Syx co-expressed in HEK293T cell lysates are shown in the right panels. Percentages from each blotted protein amount relative to “VEGF-A (−)” are shown below each lane. (E) Confirmation of the siRNA effects for GIPC1 or Syx. GIPC1 or Syx was overexpressed in HEK293T cells treated with 20 nM siControl, siGIPC1, or siSyx. The inhibitory efficiency of each siRNA on the expression of GIPC1 or Syx was compared to the siControl. The inhibitory percentages relative to the siControl are shown below each lane. (F) Colony formation assay in siNRP1, siGIPC1, or siSyx treated-DJM-1 cells in the presence or absence of exogenous VEGF-A (1 µg/ml) indicated as white columns (+) or black columns (−), respectively. Percentages from each mean relative to the siControl are shown below the graph. These data represent the means±s.d. N.S., not significant; **P<0.005; ***P<0.001.

NRP1 does not have any known signaling motif in the short 44 amino acid cytoplasmic region; therefore, it currently remains unclear whether this domain is involved in signaling. We constructed a shNRP1 vector to abrogate the expression of NRP1 in DJM-1 cells. The sequence of shNRP1 was based on siNRP1 #3, which targeted NRP1 3′UTR. shNRP1 clones (No. 12 and No. 13) did not express NRP1 and also did not support DJM-1 cell proliferation (shControl: 100%, shNRP1-12: 35%, shNRP1-13: 24%, respectively) (Fig. 4A). In subsequent experiments, we used shNRP1 clone No. 13 and infected shNRP1-DJM-1 cell clones with NRP1WT, NRP1 lacking the 44 amino acid cytoplasmic region (NRP1ΔCyto), or NRP1 lacking the C-terminus amino acids, SEA (NRP1ΔSEA) (Fig. 4B). The growth of the shNRP1 clone was less than that of the shControl clone (40% of shControl) (Fig. 4C). The lentiviral overexpression of NRP1WT restored growth, whereas NRP1ΔSEA and NRP1ΔCyto did not (shNRP1+WT: 90%, shNRP1+ΔSEA: 27%, shNRP1+ΔCyto: 23%, respectively) (Fig. 4C). These results suggested that the NRP1 cytoplasmic region, containing SEA, was essential for VEGF-A-induced proliferation.

### VEGF-A binding to NRP1 induced the interaction between GIPC1 and Syx, thereby promoting DJM-1 proliferation

GIPC1 (RGS-GAIP-interacting protein C-terminus) has a PDZ domain that interacts with the NRP1 C-terminal three amino acid residues, SEA (Wang et al., 2006). Syx has been shown to bind to the GIPC1 PDZ domain via its C-terminus (Gao et al., 2000; Garnaas et al., 2008). NRP1, GIPC1, and Syx proteins were overexpressed in HEK293T cells, which did not express VEGFR1 or VEGFR2 (supplementary material Fig. S2). A co-immunoprecipitation analysis (Co-IP) with GIPC1 (HA) showed that NRP1/GIPC1 and Syx/GIPC1 complexes were more prominent in the presence of VEGF-A (+) than in its absence (−) (Fig. 4Da, asterisks**)**. On the other hand, Co-IP with Syx showed that the GIPC1/Syx complex was increased; however, the NRP1/Syx complex was less prominent in the presence of VEGF-A (+) than in its absence (−) (Fig. 4Db, asterisks**)**. These results suggested that VEGF-A/NRP1 induced GIPC1 binding to NRP1 and formation of the GIPC1/Syx complex, which appeared to be released from NRP1.

In order to determine whether GIPC1 and Syx mediated the VEGF-A/NRP1 signal in DJM-1 cells, we treated DJM-1 cells with siGIPC1 or siSyx and analyzed proliferation in the presence of exogenous VEGF-A. The siGIPC1 and siSyx treatments both reduced the expression of GIPC1 and Syx (Fig. 4E) and inhibited the proliferation of DJM-1 cells in the absence of exogenous VEGF-A (Fig. 4F, black columns, siControl: 100%, siNRP1: 43%, siGIPC1: 15%, siSyx: 3%, respectively**)**. When exogenous VEGF-A was added, it increased the proliferation of siControl-treated DJM-1 cells (white columns, siControl: 162%). However, exogenous VEGF-A did not induce the proliferation of siNRP1-, siGIPC1-, or siSyx-treated cells (white columns, siNRP1: 60%, siGIPC1: 22%, siSyx: 13%, respectively), suggesting that GIPC1 and Syx were downstream molecules responsible for the VEGF-A/NRP1 signal that induces the proliferation of DJM-1 cells.

### Syx RhoGEF activity was important for signaling DJM-1 cell proliferation

**Fig. 5.**
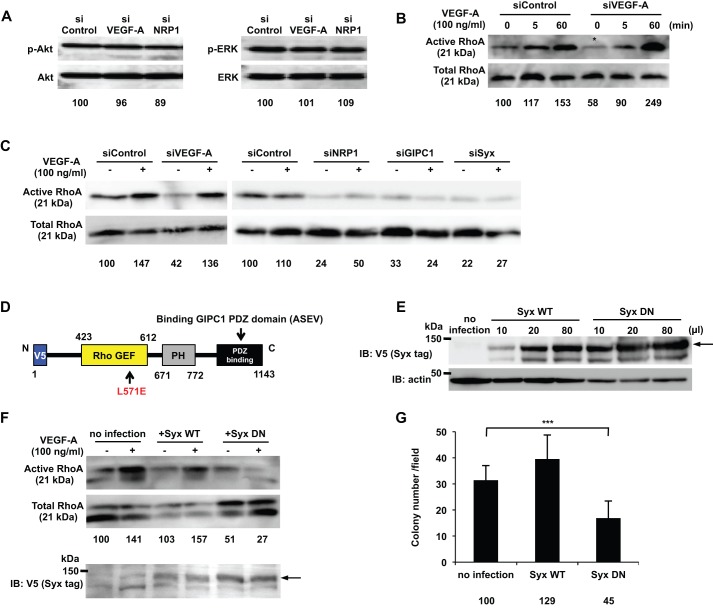
**Syx was identified as a downstream molecule of VEGF-A/NRP1 signaling and a RhoA activator that promoted DJM-1 cell proliferation.** (A) The western blot for phospho-Akt and phospho-ERK of DJM-1 cell lysates. DJM-1 cells were treated with siRNAs (siControl, siVEGF-A or siNRP1, 20 nM respectively) under anchorage-independent conditions. The same proteins were re-immunoblotted with an anti-Akt or -ERK antibody to normalize the amounts of each phospho-protein. (B,C) The RhoA activity of DJM-1 cells under anchorage-independent conditions. (B) DJM-1 cells were treated with siControl or siVEGF-A and stimulated with (+) or without (−) VEGF-A (100 ng/ml) at the indicated time points. The siVEGF-A treatment (asterisk) decreased RhoA activity below that with the siControl treatment. (C) DJM-1 cells were treated with siVEGF-A, siNRP1, siGIPC1, and siSyx in the presence of VEGF-A (+) or its absence (−). (D) Structure of dominant negative Syx (Syx DN). An amino acid substitution of Leu 571 Glu in Syx DN prevented RhoA from interacting with the mutant. The V5 epitope was tagged at the N-terminus of Syx DN. (E) Lentiviral overexpression of Syx WT or Syx DN in DJM-1 cells. Virus infection amounts were adjusted for equal expression levels of Syx WT or Syx DN in the RhoA activity assay (F) and in the colony formation assay (G). Arrow shows Syx WT or Syx DN. (F) The RhoA activity assay for Syx DN-overexpressing DJM-1 cells under anchorage-independent conditions in the presence of VEGF-A (100 ng/ml) (+) or its absence (−). A 10% input was subsequently immunoblotted with an anti-V5 antibody to normalize the amounts of each protein. The arrow shows Syx WT or Syx DN. (G) The colony formation assay for DJM-1 cells that overexpressed Syx WT or Syx DN. These data represent the means±s.d. Percentages from each mean relative to the siControl (A), siControl (−)(B,C) or no infection (F,G) are shown below the graph. ***P<0.001.

The MAPK and PI3K pathways are responsible for tumor malignancy and poor patient prognoses. The phosphorylation of MAPK (ERK) and Akt has been shown to contribute to cell proliferation and survival (Owonikoko and Khuri, 2013; Santarpia et al., 2012). However, siVEGF-A and siNRP1 did not significantly change the phosphorylation levels of either MAPK or Akt in DJM-1 cells from those in siControl cells (Fig. 5A). These results suggest that MAPK and Akt were not involved in VEGF-A/NRP1-induced DJM-1 cell proliferation.

RhoA is a regulator of cell proliferation and drives the cell cycle into the S phase (Hu et al., 1999). In siControl-treated cells, RhoA was activated in the absence of exogenous VEGF-A (Fig. 5B; 0 min; asterisk**)**. In contrast, the siVEGF-A treatment inhibited the activation of RhoA in the absence of exogenous VEGF-A (Fig. 5B, 0 min, asterisk**)**. The exogenous addition of VEGF-A activated RhoA in siControl-treated and siVEGF-A-treated cells (Fig. 5B; 5 min; 60 min**)**. All siNRP1, siGIPC1, and siSyx treatments abrogated RhoA activity in the absence of VEGF-A (−) (Fig. 5C). The exogenous addition of VEGF-A (+) restored the RhoA activity of siVEGF-A-treated cells, but not in siNRP1-, siGIPC1- and siSyx-treated DJM-1 cells (Fig. 5C), indicating that the VEGF-A/NRP1 signal induced the activation of RhoA via GIPC1 and Syx in DJM-1 cells.

RhoGEF is an activator of RhoA. The Syx RhoGEF domain is located from 423 to 612 in its amino acid sequence (Marx et al., 2005) (Fig. 5D). The amino acid residue Leu, located at 571, is important for the binding and activation of RhoA. In order to elucidate whether Syx RhoGEF activity was important for VEGF-A/NRP1-induced DJM-1 cancer cell proliferation, we constructed a lentivirus vector encoding Syx WT or a Syx mutant with a point mutation at the position of 571 Leu replaced to Glu in order to lose binding and the activation of RhoA (Marx et al., 2005). The lentiviruses of Syx WT and the Syx mutant both induced protein expression in DJM-1 cells. The infection amounts among the viruses with the different titers for protein expression were adjusted for equal expression levels in the RhoA activity assay and colony formation assay (Fig. 5E). The lentiviral overexpression of the Syx mutant interfered with the VEGF-A-induced activation of RhoA in DJM-1 cells (Fig. 5F) and inhibited DJM-1 cell proliferation (no addition: 100%, Syx WT: 129%, Syx MT: 45%) (Fig. 5G). These results suggested that Syx, the RhoGEF of RhoA, was an essential and key signaling molecule for mediating the VEGF-A-induced signal transduction that activates RhoA, leading to DJM-1 cell proliferation.

### RhoA was activated by VEGF-A/NRP1, GIPC1, and Syx to promote cancer cell proliferation

**Fig. 6.**
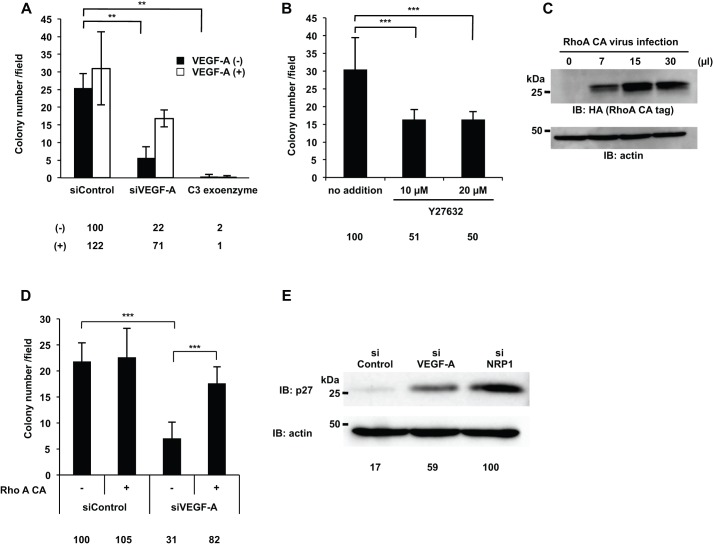
**RhoA activity was essential for the DJM-1 cell proliferation signal to induce p27^kip1^ protein degradation.** (A,B) The colony formation assay for DJM-1 cells. (A) DJM-1 cells were treated with siRNAs (siControl, siVEGF-A, 20 nM each) and C3 exoenzyme (2 μg/ml) in the presence or absence of exogenous VEGF-A (1 µg/ml), indicated as white columns (+) or black columns (−), respectively. (B) DJM-1 cells were treated with the ROCK inhibitor, Y27632 (10 or 20 μM). (C) An increase in the lentiviral infection of RhoA constitutively active form (RhoA CA) enhanced the RhoA active form in DJM-1 cells. (D) The colony formation assay for siRNAs (siControl or siVEGF-A, 20 nM each) treated-DJM-1 cells with (+) or without (−) RhoA CA expression. Percentages from each relative to the siControl (−)(A,D) or no addition (B) are shown below the graph. (E) DJM-1 cells were treated with siControl, siVEGF-A, or siNRP1 (20 nM each) under anchorage-independent conditions and total cell lysates were immunoblotted with an anti-p27 antibody. Percentages from the p27 level in each siRNA treated-cell lysate relative to siNRP1 are indicated below the lane. The same proteins were re-immunoblotted with an anti-actin antibody to normalize the amounts of each protein. These data represent the means±s.d. **P<0.005; ***P<0.001.

In order to determine whether the activation of RhoA promoted DJM-1 cell proliferation, DJM-1 cells were treated with C3 exoenzyme, a specific inhibitor of RhoA. C3 exoenzyme completely suppressed DJM-1 cell proliferation in the absence and presence of exogenous VEGF-A (2% and 1% of siControl, respectively) (Fig. 6A). Y27632, a ROCK inhibitor that is a downstream effector of RhoA, suppressed DJM-1 cell proliferation (no addition: 100%, 10 µM: 51%, 20 µM: 50%, respectively) (Fig. 6B). Proliferation was recovered (31% to 82%) when RhoA constitutively active form (RhoA CA) was overexpressed in siVEGF-A-treated DJM-1 cells (Fig. 6C,D). p27 was degraded by the activation of RhoA, thereby leading to cell proliferation (S-phase entry). p27 is an inhibitor of G1 cyclin-dependent kinase and regulates cell proliferation downstream of RhoA. Under anchorage-independent conditions, the accumulation of p27 was greater in siVEGF-A- and siNRP1-treated DJM-1 cells than in siControl-treated DJM-1 cells (Fig. 6E). Taken together, these results demonstrated that VEGF-A/NRP1 signaling activated RhoA activity via a GIPC1/Syx complex to inhibit the accumulation of p27.

### The oligopeptide that inhibited GIPC1 and Syx interactions suppressed RhoA activation and DJM-1 proliferation

**Fig. 7.**
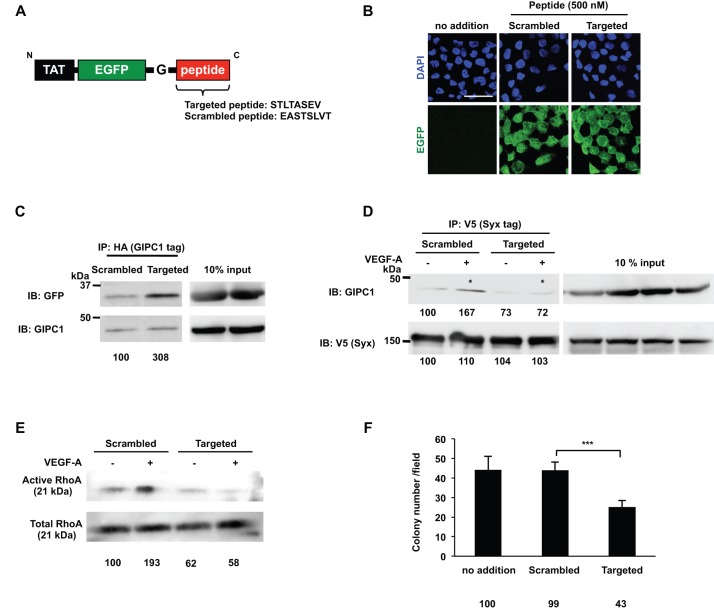
**The oligopeptide that inhibited the GIPC1 and Syx interaction suppressed RhoA activity and the proliferation of DJM-1 cells.** (A) A schematic of the construct that contained TAT, EGFP, and the Gly insertion prior to the Targeted peptide sequence (STLTASEV; Syx C terminus sequence). The Scrambled peptide amino acid sequence is also shown in the lower case (EASTSLVT). (B) Confirmation of the peptide incorporation into DJM-1 cells. DJM-1 cells were treated with the Scrambled or Targeted peptide (500 nM each) for 1 h. Confocal images indicated the Scrambled or Targeted peptide in the intracellular region of DJM-1 cells (green). Nuclei in the same position were shown in the upper panels (blue). Scale bar: 30 μm. (C) The co-immunoprecipitation assay with the Target peptide. HA-tagged GIPC1 was overexpressed in HEK293T cells. The Scrambled or Targeted peptide was mixed with the cell lysate and co-immunoprecipitated with GIPC1 after a 1 h rotation at 4°C. The same lysates (10% input) were immunoblotted with anti-GFP or anti-GIPC1 antibodies to normalize the amounts of the peptide and GIPC1. Percentages from each relative to the Scrambled are shown below the graph. (D) NRP1, GIPC1, and Syx vectors were transfected and expressed in HEK293T cells, which were subsequently treated with the Targeted or Scrambled peptide for 16 h. After a 10 min stimulation with (+) or without (−) VEGF-A (100 ng/ml), the cells were lysed and the indicated proteins in the cell lysates were co-immunoprecipitated with V5-tagged Syx (left panels). VEGF-A induced the GIPC1/Syx interaction in the presence of the Scrambled peptide (asterisk). On the other hand, the Targeted peptide abrogated the GIPC1/Syx interaction (asterisk). Percentages from each protein level [GIPC1 or V5 (Syx)] compared to the lane of Scrambled (−) are indicated below the lane. The same lysates (10% input) were immunoblotted with anti-GIPC1 or V5 antibodies to normalize the amounts of each protein. (E) The RhoA activity assay. DJM-1 cells were treated with the Targeted or Scrambled peptide and stimulated with (+) or without (−) VEGF-A (100 ng/ml) under anchorage-independent conditions. The same lysates (10% input) were immunoblotted with an anti-RhoA antibody to normalize the protein amounts with each treatment. Percentages from each relative to the Scrambled (−) are shown below the graph. (F) The colony formation assay. DJM-1 cells were treated with 500 nM of the Targeted or Scrambled peptide. The Targeted peptide inhibited DJM-1 cell proliferation, whereas the Scrambled peptide did not. These data represent the means±s.d. Percentages from each mean relative to the no addition control are shown below the graph. ***P<0.001.

We designed a membrane-penetrating peptide targeted to inhibit complex formation between GIPC1 and Syx (Fig. 7A). The 30 kDa targeted peptide consisted of TAT, a cell penetrating sequence of the HIV virus, EGFP, and eight amino acid residues that included the Syx C-terminal amino acid sequence (STLTASEV). The Syx C-terminal amino acid sequence was important for recognizing the GIPC1 PDZ domain in the GIPC1/Syx interaction; therefore, the targeted peptide acted as a competitive inhibitor. The incorporation of these peptides into DJM-1 cells was confirmed through the detection of a green fluorescent protein linked to the peptides after a 1 h treatment (Fig. 7B). In order to establish whether the Targeted peptide interacted with GIPC1, HA-tagged GIPC1 was overexpressed in HEK293T cells and the cell lysate was incubated with either the Scrambled peptide or Targeted peptide. Binding of the Targeted peptide with GIPC1 was 3-fold greater than that with the Scrambled peptide (Fig. 7C). In order to evaluate whether the Targeted peptide inhibited the interaction between GIPC1 and Syx, NRP1, GIPC1, and Syx vectors were transfected and expressed in HEK293T cells and these cells were then treated with the Targeted or Scrambled peptide for 16 h. After a 10 min stimulation with (+) or without (−) VEGF-A (100 ng/ml), the cells were lysed and the indicated proteins in the cell lysates were co-immunoprecipitated with V5-tagged Syx (left panels). VEGF-A/NRP1 induced GIPC1/Syx complex formation in the presence of the Scrambled peptide (Fig. 7D, asterisk). On the other hand, the Targeted peptide abrogated the VEGF-A/NRP1 signal-induced GIPC1/Syx interaction (Fig. 7D, asterisk**)**. In addition, the Targeted peptide more strongly prevented the activation of RhoA than the Scrambled peptide in the absence and presence of VEGF-A (Fig. 7E). Additionally, DJM-1 cell proliferation was inhibited by the Targeted peptide (Scramble peptide: 99%, Targeted peptide: 43%) (Fig. 7F). These results demonstrated that, in the VEGF-A/NRP1 signaling pathway, the GIPC1 and Syx interaction was necessary for the activation of RhoA in order to promote the proliferation of cancer cells.

## DISCUSSION

**Fig. 8.**
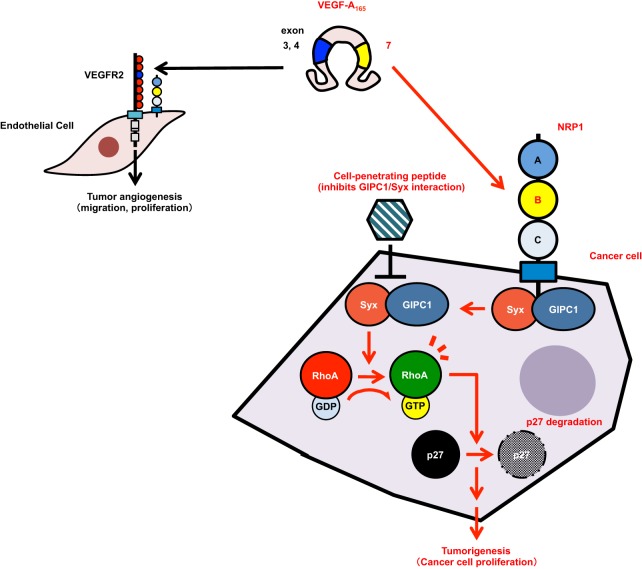
**Schematic of the VEGF-A/NRP1 signaling pathway to promote cancer cell proliferation.** Schematic of a new pathway of NRP1 signals leading to the proliferation of cancer cells. Cancer cell-secreted VEGF-A binds to NRP1 in an autocrine manner and stimulates complex formation by GIPC1, a scaffold protein, and Syx, RhoGEF, via the NRP1 cytoplasmic region. The GIPC1/Syx complex increases the activation of RhoA in order to induce the degradation of p27 and consequently promotes cancer cell proliferation.

In the present study, VEGF-A induced the cancer cell proliferation of PC3M (prostate cancer), DJM-1 (skin cancer), and U87MG (glioblastoma cell) in an anchorage-independent manner via the NRP1 signaling pathway (Fig. 8). The knockdown of VEGF-A or NRP1 abrogated the proliferation of these cancer cells. We selected skin cancer-derived DJM-1 cells, which only express NRP1 as the VEGF-A receptor and grow faster than other cancer cells under anchorage-independent conditions.

The NRP1 structure governs its bioactivity. The A domain mostly binds semaphorins, whereas the B domain binds VEGF-A (Geretti et al., 2008). The treatment of cancer cells with the soluble NRP1 B domain or siNRP1 inhibited proliferation in an anchorage-independent manner. Stable shNRP1-DJM-1 clones also decreased proliferation. Taken together, these results indicated that endogenous NRP1 transduced a VEGF-A proliferative signal. PlGF, a member of the VEGF-A family, activated the MAPK pathway via NRP1 in medulloblastoma (Snuderl et al., 2013). NRP2, an isoform of NRP1, was previously shown to transduce the activation of Akt in pancreatic cancer cells (Dallas et al., 2008). However, in the present study, these pathways were not involved in VEGF-A/NRP1 signaling in DJM-1 cells because ERK and Akt phosphorylation levels did not change even when the expression of VEGF-A or NRP1 was decreased by siRNA. The NRP1 C-terminal 3 amino acids (SEA), which contribute to recognition of the PDZ domain of GIPC1, were of particular interest in the present study. The SEA motif was previously shown to be critical for binding GIPC1 (Wang et al., 2006). In the present study, lentiviral infection of the two NRP1 cytoplasmic deletion mutants (NRP1ΔSEA or NRP1ΔCyto) into the shNRP-DJM-1 clone failed to induce anchorage-independent growth in response to VEGF-A. VEGF-A increased the GIPC1 interaction with NRP1, indicating that the NRP1/GIPC1 interaction is necessary for stimulating DJM-1 cell proliferation. GIPC1 has been suggested to play an important role in cancer cell proliferation. GIPC1 was shown to bind to IGF-1R via its PDZ domain in order to promote pancreatic cancer cell proliferation (Muders et al., 2009). By binding VEGF-A to NRP1, GIPC1 mediates the interaction between NRP1 and ABL1, which activates tyrosine kinase activity and associates with integrins, leading to induce tumor growth (Goel and Mercurio, 2013). Syx is a RhoGEF that stimulates RhoA activity (Garnaas et al., 2008). The molecular interaction between GIPC1 and Syx was identified using two-yeast hybridization (Gao et al., 2000). Syx has been implicated in tumorigenesis, brain tumors, and neuroblastoma (De Toledo et al., 2001). siSyx inhibited the proliferation of DJM-1 cells, indicating that Syx is involved in a signaling pathway that promotes cell proliferation.

Previous studies reported that NRPs required an interaction with co-receptors such as VEGFR1 or VEGFR2 to induce signals for cell survival and migration (Kawamura et al., 2008). Favier et al. demonstrated that NRPs associated with VEGFR2 in ligand-dependent and ligand-independent manners in HEK293T cells that transiently overexpressed NRPs and VEGFR2, thereby inducing VEGFR2 phosphorylation (Favier et al., 2006).

In the present study, we showed that the VEGF-A/NRP1 signaling pathway promoted cancer cell proliferation even though cancer cells do not express VEGFR1 or VEGFR2 (Fig. 8). We used HEK293T cells that expressed NRP1, but not VEGFR2 in co-immunoprecipitation to analyze interactions between NRP1/GIPC1 or GIPC1/Syx (supplementary material Fig. S2). As a consequence, VEGF-A induced NRP1/GIPC1 and GIPC1/Syx complex formation without the expression of VEGFR1 or VEGFR2. These results indicated that VEGF-A signals via NRP1 in a VEGFR1- or VEGFR2-independent manner, resulting in cancer cell proliferation.

RhoA is a small GTPase that drives the cell cycle into the S-phase with the degradation of p27. p27 protein levels were previously reported to be reduced in many organ sites in the majority of human malignancies (Hu et al., 1999). The activation of RhoA is known to induce the protein degradation of p27^Kip1^. The “dominant negative” effect of the Syx mutant (Syx DN) on RhoA suggests that Syx is involved in the VEGF-A/NRP1 signaling pathway. RhoA has been implicated in virtually all stages of cancer progression. The treatment of DJM-1 cells with the RhoA-specific inhibitor, C3 exoenzyme or ROCK inhibitor (Y27632) suppressed DJM-1 cell proliferation. The knockdown of VEGF-A or NRP1 upregulated the expression of the p27 protein. In addition, the overexpression of constitutively active RhoA in siVEGF-A-treated cells rescued the inhibition of proliferation, indicating that endogenous VEGF-A-binding NRP1 activated Syx RhoGEF in order to stimulate the activation of RhoA, thereby leading to the degradation of p27. Taken together, these results suggested that endogenous VEGF-A/NRP1 signaling in DJM-1 cells induced the degradation of p27 in order to constitutively stimulate progress into the S-phase.

The importance of the molecular mechanism by which the GIPC1 interaction with Syx activates Syx GEF activity was demonstrated in the present study. As a novel tactic, we herein generated a peptide that contained the HIV TAT sequence, which enabled the peptide to penetrate the cell membrane and inhibited complex formation by GIPC1 and Syx (Fig. 8). This peptide consisted of eight amino acids corresponding to the sequence of Syx C-terminus (STLTASEV). The C-terminal five or six amino acids of a binding partner of GIPC1 were shown to lead to sufficient affinity for binding for the PDZ domain (Muders et al., 2009). This study showed that the peptide abrogated the complex formation necessary for Syx RhoGEF activation. Syx is known to include an auto-inhibitory domain in the C-terminal region. A hypothetical mechanism has been proposed by which GIPC1 binding to the auto-inhibitory domain in the C-terminal region of Syx triggers RhoGEF activity.

In conclusion, this study provided evidence for a new pathway of VEGF-A/NRP1 signaling leading to the proliferation of cancer cells. Furthermore, we showed that the molecular function of GIPC1 and its interaction with Syx played a key role in the activation of RhoA, which induced the degradation of p27. The inhibition of VEGF-A/NRP1 signaling may represent a new strategy against cancer and be applied in the design of new cancer drugs.

## MATERIALS AND METHODS

### Reagents

Recombinant human-VEGF-A_165_, VEGF-A_121_, and PlGF-2 were purchased from R&D Systems (Minneapolis, MN, USA). The VEGFR kinase inhibitor SU5614 was purchased from Merck (Whitehouse Station, NJ, USA). The RhoA-specific inhibitor, C3 exoenzyme, was purchased from Cytoskeleton (Denver, CO, USA) and Y27632 was purchased from EMD Millipore (Billerica, MA, USA). The anti-VEGF-A antibody, Avastin^®^ (bevacizumab), was kindly provided by Dr Mark Kieran (Dana-Farber Cancer Institute, Boston, MA, USA).

### Animal studies

All animal experiments were performed in accordance with Kyoto Sangyo University's animal experiment guidelines. DJM-1 cells (4×10^6^ cells per 100 µl HBSS) were orthotopically inoculated at the right flank of 6-week-old female BALB/C Slc-nu/nu mice (SHIMIZU Laboratory Supplies Co., Ltd., Sakyo-ku Kyoto, Japan). After 2 weeks, mice were sacrificed and tumors were isolated and embedded in OCT compound (SAKURA Tissue Teck, Koto-ku, Tokyo, Japan).

### Cell culture and transfection

The human skin cancer line, DJM-1 was kindly provided by Dr H. Katayama (Katayama et al., 1989) (Katayama Clinic, Maebashi, Japan) and cultured in DMEM supplemented with 10% fetal bovine serum (FBS) and glucose (final 4.5 mg/ml). HEK293T cells were purchased from ATCC and cultured in DMEM supplemented with 10% FBS. PC3M cells and U87MG cells were purchased from ATCC and cultured in RPMI-1640 supplemented with 10% FBS for PC3M cells. U87MG cells were cultured in EMEM supplemented with 10% FBS. Human Umbilical Vein Endothelial Cells (HUVEC) were purchased from LONZA (Gampel, Valais, Switzerland) and maintained in endothelial cell growth medium (EGM-2).

The transfection of expression vectors into HEK293T was performed with FuGENE6 (Promega, Madison, WI, USA). siLentFect™ reagents (Bio-Rad, Hercules, CA, USA) were used for all siRNA treatments as directed in the instruction manual.

### Antibodies

The following primary antibodies were used: GIPC1 (N-19) goat; neuropilin-1 (C-19) goat; neuropilin-2 (C-9) mouse; PLEKHG5 (KB-7) mouse; Flt-1 (C-17) rabbit and Flk-1 (C-1158) rabbit antibodies (Santa Cruz, Dallas, TX, USA); Akt (pan) (C67E7) rabbit; phospho-Akt (Ser473) (D9E) XP™ rabbit; p44/42 MAPK (Erk1/2) rabbit; phospho-p44/42 MAPK (Erk1/2) (Thr202/Tyr204) rabbit; neuropilin-1 (D62C6) rabbit and RhoA (67B9) rabbit antibodies (Cell Signaling Technology, Danvers, MA, USA). An anti-HA 11 clone (16B12) mouse antibody was purchased from Covance (Princeton, NJ, USA). An anti-V5 rabbit antibody was purchased from Bethyl (Montgomery, TX, USA). An anti-actin rabbit antibody (Cat: A2013) was purchased from Sigma-Aldrich (St. Louis, MO, USA).

The secondary antibodies used were: horseradish peroxidase (HRP)-conjugated anti rabbit IgG; HRP-conjugated anti goat IgG; a HRP-conjugated anti-mouse IgG antibody (Jackson Immuno Research, West Grove, PA, USA). DAPI was purchased from Invitrogen (Life Technologies, Van Allen Way, Carlsbad, CA, USA). A biotin-conjugated rat IgG antibody was purchased from VECTOR (Burlingame, CA, USA).

### Plasmids

Human NRP1WT, HA-tagged GIPC1, V5-tagged Syx, HA-tagged constitutively active RhoA, and soluble NRP constructs were inserted using the pcDNA 3.1 TOPO expression vector (Life Technologies). HALO-tagged Syx was purchased from the Kazusa DNA Research Institute (KIAA0720) and used as a template to generate the V5-tagged Syx construct. The human NRP1ΔSEA construct was generated by PCR using NRP1WT as a template and primers that introduced a *Not*I or *Bam*HI restriction site were inserted into the pcDNA 3.1 TOPO expression vector.

Forward primer: 5′-GGGCGGCCGCACCACCATGGAGAGGGGGCTGCCGCTCCTC-3′, reverse primer: 5′-GGGGATCCTCATGCCTCCGAATAAGTACTCT-3′.

Syx WT and a dominant negative mutant were generated by point mutations using the following primers; Forward primer: 5′-CCAAGTACCCGCTGGAGCTCAAGTCGGTGC-3′, reverse primer: 5′-GCACCGACTTGAGCTCCAGCGGGTACTTGG-3′.

PCR products were digested with *Not*I and *Bam*HI and inserted into the pcDNA 3.1 expression vectors.

NRP1WT, NRP1ΔSEA, and NRP1ΔCyto lentiviruses were based on the NRP1 pcDNA 3.1 construct and generated by PCR using the following primers that introduced *Not*I and *Bam*HI restriction sites. The same primer for all NRP1 constructs was used as the forward primer and PCR products were subcloned into the pHAGE lentiviral backbone vector as described above (Shimizu et al., 2008).

Forward primer: 5′-GGGCGGCCGCGCCACCATGGAGTGGGGGCTGCCGCTC-3′, reverse primers; WT: 5′-CCGGATCCCTCTGTCTGCCTTCATGCCTC-3′, ΔSEA: 5′-AAGGATCCTCAATAAGTACTCTGTGTATTCAGTTTGTC-3′ and ΔCyto: 5′-GGGGATCCTCAGTACAGCAC GACCCCACAGAC-3′.

Syx WT or the DN-V5 tagged lentivirus was based on each pcDNA 3.1 construct and generated by PCR using the following primers that introduced the *Not*I or *Bam*HI restriction site. PCR products were subcloned into the pHAGE lentiviral backbone vector.

Forward primer: 5′-GCGGCCGCGCCACCATGGGTAAGCCTATCCCTAACCCTCTCC TCGGTCTCGATTCTACGGGTGACGAGACCAGAGCCCCGCT-3′, reverse primer: 5′-GGGGGATCCTCAGACCTCCGAGGCAGTGAGC-3′.

The following NRP1 shRNA sequences based on siNRP1 #3 were inserted into pSilencer™ 4.1-CMV neo (Ambion; Life Technologies); sense primer: 5′-GATCCCGGGCTGAGGATTGTACAGTTCAAGAGACTGTACAATCCTCAGCCCGTCA-3′, antisense primer: 5′-AGCTTGACGGGCTGAGGATTGTACAGTCTCTTGAACTGTACAATCCTCAGCCCGG-3′.

### Preparation of Lentivirus vectors of NRP1 WT and mutants

Each NRP1WT, NRP1ΔSEA, and NRP1ΔCyto in the pHAGE lentiviral backbone vector was co-transfected with the helper plasmids (tat, rev, gag-pol and VSV-G) to HEK293 cells as described previously (Shimizu et al., 2008). Viral supernatants were assembled and concentrated at 38,000×***g*** for 1.5 h at 4°C. The collected virus was infected with 10 µg/ml polybrene (Millipore) to express NRP1WT and the mutants in DJM-1 cells.

### siRNAs

siGENOME smart pool control siRNA (D-001206), GIPC1 siRNA (M-019997), and Syx siRNA (M-013873) were purchased from Dharmacon RNAi Technologies (Thermo Scientific, Waltham, MA, USA). Human VEGF-A siRNA #1, #2, and #3 were annealed using the following sequences, respectively; VEGF-A siRNA #1; sense primer: 5′-GCAUUGGAGCCUUGCCUUGCUTT-3′, antisense primer: 5′-AGCAAGGCAAGGCUCCAAUGCTT-3′. VEGF-A siRNA #2; sense primer: 5′-GGAGCCUUGCCUUGCUGCUCUTT-3′, antisense primer: 5′-AGAGCAGCAAGGCAAGGCUCCTT-3′. VEGF-A siRNA #3; sense primer: 5′-GGACCUAUGUCCUCACACCTT-3′, antisense primer: 5′-GGUGUGAGGACAUAGGUCCTT-3′.

Human NRP1 siRNA #1, #2, and #3 were annealed using the following sequences, respectively; NRP1 siRNA #1; sense primer: 5′-AAUCAGAGUUUCCAACAUATT-3′, antisense primer: 5′-UAUGUUGGAAACUCUGAUUTT-3′. NRP1 siRNA #2; sense primer: 5′-GUGGAUGACAUUAGUAUUATT-3′, antisense primer: 5′-UAAUACUAAUGUCAUCCACTT-3′. NRP1 siRNA #3; sense primer: 5′-GACGGGCUGAGGAUUGUACTT-3′, antisense primer: 5′-GUACAAUCCUCAGCCCGUCTT-3′.

### shNRP1 construction and transfection

The designed shNRP1 oligonucleotide sequences were based on siNRP1 #3. Sense oligo: 5′-GATCCCGGGCTGAGGATTGTACAGTTCAAGAGACTGTACAATCCTCAGCCCGTCA-3′, antisense oligo: 5′-AGCTTGACGGGCTGAGGATTGTACAGTCTCTTGAACTGTACAATCCTCAGCCCGG-3′. The sense and antisense oligonucleotides were annealed and inserted at the *Bam*HI and *Hin*dIII restriction sites into the pSilencer™ 4.1-CMV neo plasmid (Ambion; Life Technologies). DJM-1 cells were transfected with the shNRP1 construct or control plasmid by electroporation with a 0.4 cm cuvette (GenePulser Xcell; Bio-Rad). The transfectants were screened in 400 µg/ml G418-contained growth medium to obtain stable DJM-1 cell clones (shNRP1 clone #12 and #13, shControl).

### Peptides

The expression plasmids for the fusion proteins, TAT-EGFP-peptide 1 (STLTASEV) and TAT-EGFP-scramble 1 (EASTSLVT) were prepared by the site-directed mutagenesis of DNA sequences encoding TAT-EGFP cloned in a pGEX-6P-3 expression vector (GE Healthcare Life Sciences, Buckinghamshire, UK) (Kizaka-Kondoh et al., 2009). DNA primers for the amplification of plasmids were as follows: for TAT-EGFP-peptide 1, 5′-GCCAGCGAGGTGTAAATCGTGACTGACTGACGATCTGCC-3′ and 5′-GGTCAGGGTGCTGCCCTTGTACAGCTCGTCCATGGCG-3′; for TAT-EGFP-scramble 1, 5′-AGCCTGGTGACCTAAATCGTGACTGACTGACGATCTGCC-3′ and 5′-GGTGCTGGCCTCGCCCTTGTACAGCTCGTCCATGGCG-3′. The resultant plasmids were introduced into BL21-CodonPlus (DE3) cells (Agilent Technologies, Santa Clara, CA, USA). Fusion proteins were expressed as glutathione S-transferase (GST)-tagged proteins and purified by affinity chromatography, as previously described (Kizaka-Kondoh et al., 2009). The GST-tag was removed, and final proteins were equilibrated in PBS.

### Immunoprecipitation (IP)

HEK293T cells were transfected with NRP1WT, GIPC1, and Syx plasmids with FuGENE6. The cells were stimulated with or without 100 ng/ml VEGF-A for 15 min and were lysed with RIPA buffer (1% NP-40, 0.5% Sodium deoxycholate, 0.1% SDS, NaCl 100 mM, Tris-HCl 50 mM, pH7.4). The cell lysates were incubated with the anti-HA, anti-Syx, or anti-V5-antibody at 4°C overnight. Protein G Sepharose beads (Protein G Sepharose 4 Fast Flow, GE Healthcare) were added and rotated at 4°C for 1.5 h. After washing the beads with cold RIPA buffer, proteins were removed from the beads in 40 μl 2× Laemmlli Sample buffer and analyzed by SDS-PAGE and western blotting. In the input analysis, 1/10 volume of the cell lysate was used.

### Western blotting

Cells were lysed with cold RIPA buffer. After running SDS-PAGE, proteins were transferred to a PVDF membrane (Millipore) and blotted with primary antibody-diluted 4% skim milk in TBST at 4°C overnight followed by incubation with a HRP-conjugated secondary antibody. The blots were treated with chemiluminescent substrate solution (Thermo Fisher Scientific, Waltham, MA, USA) and exposed to LAS-4000 mini (Fujifilm Co., Tokyo, Japan) to reveal immunoreactive bands. Percentages from each band on densitometry compared to the control were indicated in the lower lanes in the figures. The western blot analysis was repeated 3 times.

### VEGF-A ELISA

A human VEGF Quantikine^®^ ELISA kit (R&D Systems) was used. DJM-1 cells were seeded at a density of 2×10^5^ cells/well/6-well plate, followed by a treatment with 20 nM siRNA. The medium was changed to DMEM containing 1% BSA, and cells were incubated for 3 days at 37°C in 5% CO_2_. The conditioned media were diluted to ten-fold with serum free-DMEM, and VEGF-A levels were measured using the manufacturer's protocol. The measurement of VEGF-A concentrations from each sample was duplicated and the ELISA experiment was repeated twice.

### Soft agar assay

DJM-1, PC3M, or U87MG cells were treated with 20 nM siRNA or infected with a lentivirus before being seeded in agar. Two milliliters of growth medium containing 0.72% agar was prepared in a 35-mm dish and solidified as bottom agar. Cells (DJM-1: 5×10^4^ cells, PC3M and U87MG: 1×10^5^ cells) were suspended in 2 ml of culture medium containing 0.36% agar and, after the addition of ligands or chemicals, layered on the bottom agar. Two weeks later, viable cells were stained with 300 µg/ml 3-(4,5-Dimethyl-2-thiazolyl)-2,5-diphenyl-2H-tetrazolium bromide (MTT) solution. Colony diameters were analyzed by Image J software and the numbers of colonies larger than 80 µm in diameter were counted per 6 to 7 microscopic fields. The means±s.d of colony numbers are shown. Percentages from each mean compared to the control are indicated below the graphs in the figures. Each experiment was repeated at least two or three times.

### HUVEC migration assay

A migration assay was performed for HUVEC using Transwell inserts with a pore size of 8.0 μm (Corning, NY, USA). Membranes were coated with 0.1% gelatin. The conditioned medium of DJM-1 cells was prepared with a siRNA treatment, cultured in 2% FBS-EBM-2 for 72 h, and placed into the bottom chamber. Five thousand HUVEC were suspended in 2% FBS-EBM-2 medium, seeded into the upper compartments, and cultured for 16 h. Migrated cells were stained with Diff-Quick. The stained cells in 6 microscopic fields were counted.

### RhoA activity assay

A RhoA activity assay was performed and quantified using the RhoA activation assay kit according to the manufacturer's instructions (Cytoskeleton, Denver, CO, USA). Cells were treated with 20 nM siRNA before use and were seeded in 5 mg/ml polyHEMA (poly 2-hydroxyethyl methacrylate; Sigma)-coated 100-mm dishes for cultivation under anchorage-independent conditions overnight (Fukazawa et al., 1995). Cells were stimulated with 100 ng/ml VEGF-A_165_ for 10 min or the indicated time. Cells were lysed with Lysis buffer. The clarified cell lysate was incubated with Rhotekin-RBD protein agarose beads and rotated at 4°C for 90 min. The beads were washed with wash buffer and denatured. Active RhoA was detected by western blotting.

### Immunohistochemistry

DJM-1 tumors were frozen and sliced to a thickness of 10 µm using Leica CM3050 S (Leica, Wetzlar, Germany). The frozen sections were rehydrated with TBST and fixed with 100% methanol at −20°C for 20 min. In order to inactivate endogenous peroxidase activity, sections were incubated in 0.3% H_2_O_2_ solution containing 1% sodium azide overnight at room temperature. These sections were washed with TBST and blocked with 3% BSA/3% house serum and 1% sodium azide in TBST for 40 min, followed by incubation with an anti-mouse CD31 antibody diluted with blocking buffer (1/200) overnight. The sections were then incubated with biotin-conjugated anti-rat IgG for 1 h and developed with an ABC kit (Vector Labs, Burlingame, CA, USA) according to the manufacturer's instructions. Sections were counterstained with hematoxylin (Wako Chemicals Co., Kyoto, Japan). Photographs were taken with NIS-Elements (Nikon, Tokyo, Japan).

### Statistical analysis

Differences in means among treatments in the colony formation assay, migration assay and ELISA were evaluated with the Student's *t*-test: **P<0.005; ***P<0.001; N.S, not significant.

## Supplementary Material

Supplementary information
