# Supplementary material for: VEGF-A/NRP1 stimulates GIPC1 and Syx complex formation to promote RhoA activation and proliferation in skin cancer cells
Source: Biol Open. 2015 Jul 24;4(9):1063–76. doi: 10.1242/bio.010918 (PMC4582117; doi:10.1242/bio.010918)
Supplement: Supplementary information [file supp_4_9_1063__index.html]

VEGF-A/NRP1 stimulates GIPC1 and Syx complex formation to promote RhoA activation and proliferation in skin cancer cells — Supplementary information 

# VEGF-A/NRP1 stimulates GIPC1 and Syx complex formation to promote RhoA activation and proliferation in skin cancer cells

## BIO010918 Supplementary information

**Files in this Data Supplement:**

- Supplementary information
