# Supplementary material for: VEGF-A/NRP1 stimulates GIPC1 and Syx complex formation to promote RhoA activation and proliferation in skin cancer cells
Source: Biol Open. 2015 Jul 24;4(9):1063–76. doi: 10.1242/bio.010918 (PMC4582117; doi:10.1242/bio.010918)
Supplement: Supplementary information [file supp_bio.010918_BIO010918supp.pdf]

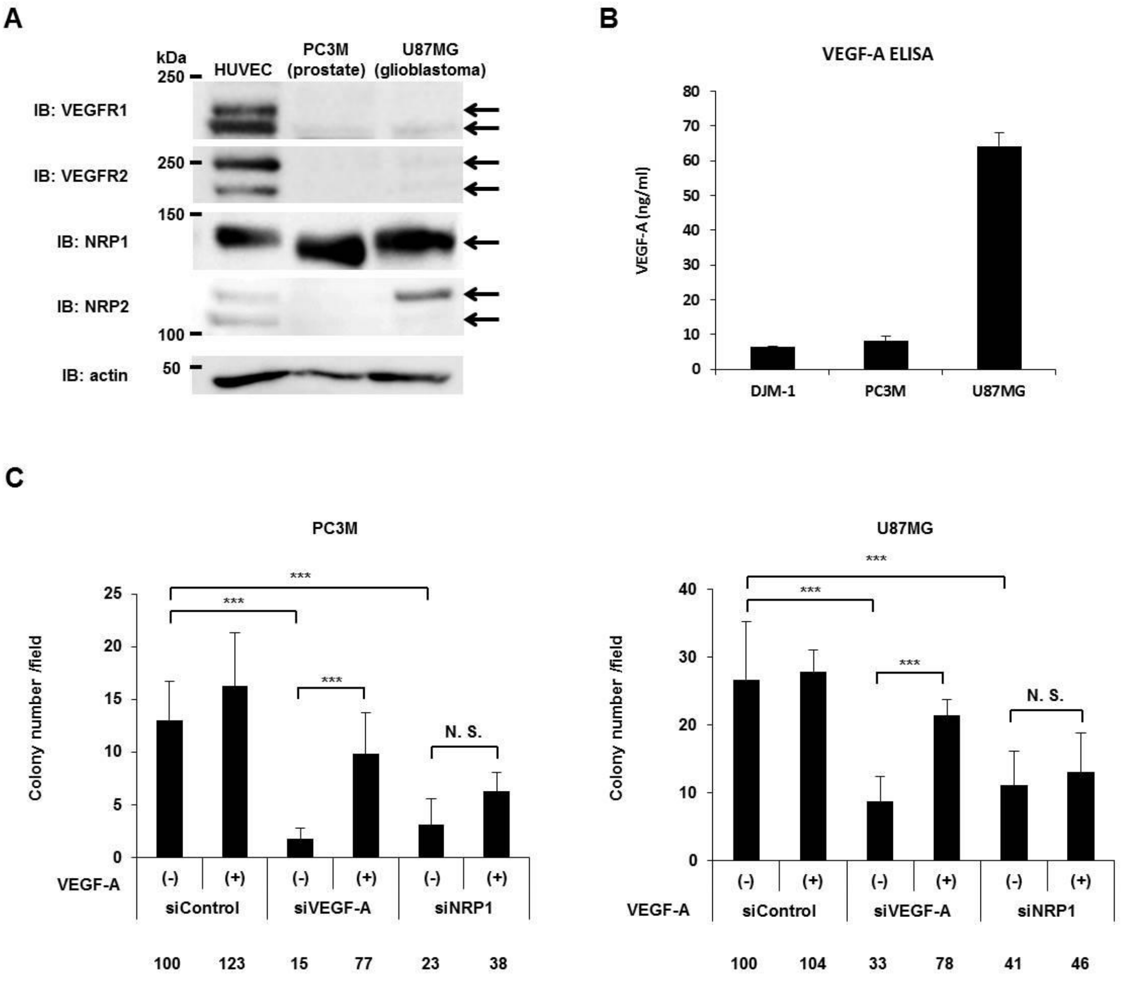

Fig. S1. VEGF-A/NRP1 signaling pathway promoted PC3M and U87MG cells proliferation. Cancer cells were seeded into 35 mm culture dish for 8×10^5 cells. Culture for over night, changed medium to 1 % BSA contained serum free medium and cultured to 72 h. (A) The NRP1 protein (~130 kDa) was strongly expressed in PC3M and U87MG. All cell lines expressed NRP1, but did not express VEGFRs. U87MG cells expressed NRP1 and NRP2. (B) U87MG cells secreted the highest levels of VEGF-A into conditioned medium. (C) The siVEGF-A or siNRP1 treatment inhibited the proliferation of PC3M (siControl: 100%, siVEGF-A: 15%, siNRP1: 23%) and U87MG cells (siControl: 100%, siVEGF-A: 33%, siNRP1: 41%). The addition of exogenous VEGF-A rescued the proliferation of siVEGF-A-treated cells (PC3M: 77%, U87MG: 78%). In contrast, the addition of VEGF-A did not recover the proliferation of siNRP1-treated cells (PC3M: 38%, U87MG: 46%), suggesting that NRP1 mediated VEGF-A signaling to induce PC3M and U87MG cell proliferation as in DJM-1 cells.

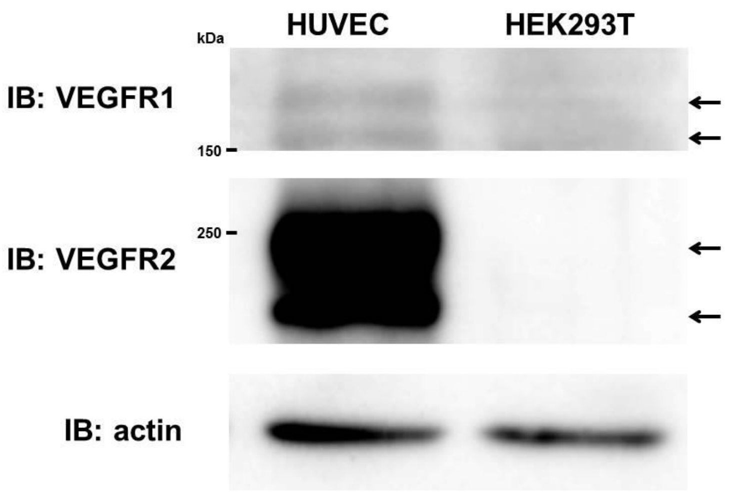

Fig. S2. HEK293T cells did not express VEGFR1 and VEGFR2.

Biology Open • Supplementary information
